# CHK1 inhibition overcomes gemcitabine resistance in non-small cell lung cancer cell A549

**DOI:** 10.1080/23723556.2025.2488537

**Published:** 2025-04-09

**Authors:** Zhi-Yin Ke, Tian Fu, Xue-Chun Wang, Xuan Ma, Hai-Han Yin, Wen-Xuan Wang, Yong-Jun Liu, Ai-Ling Liang

**Affiliations:** a Department of Biochemistry and Molecular Biology & Department of Clinical Biochemistry, Guangdong Provincial Key Laboratory of Medical Immunology and Molecular Diagnosis, Guangdong Medical University, Dongguan, China; b Department of Clinical Laboratory, Zhanjiang Central Hospital, Zhanjiang, China; c Department of Clinical Laboratory, Xinle City Hospital, Shijiazhuang, China

**Keywords:** Non-small cell lung cancer, A549 cells, drug resistance, gemcitabine, CHK1

## Abstract

The purpose of the study is mainly to investigate anti proliferation of non-small cell lung cancer A549 cells and its mechanism by inhibition of CHK1 expression combined with gemcitabine. The mRNA and protein levels of genes were analyzed by RT-qPCR and Western blot, respectively. Cell viability was detected by CCK-8 assay and clone formation assay. The detection of the cell cycle was used by Annexin V/7-amino-actinomycin D apoptosis detection kit. Analysis of DNA damage was done by immunofluorescence and alkaline comet assay. The results showed that inhibition of CHK1 and gemcitabine combination significantly reduced the proliferation ability of the two cell lines. We also revealed the degradation of full-length PARP and reduced Bcl-2/Bax ratio on increased apoptosis. Inhibition of CHK1 expression leads to DNA damage, induces phosphorylation of γ-H2AX, and affects the repair of homologous recombination ability through Rad51. Mechanistically, gemcitabine increased phosphorylation-ATR and phosphorylation-CHK1, indicating activation of the DNA repair system and ATR-CHK1-CDC25A pathway. Inhibition of CHK1 resulted in increased synthesis of CDK2/Cyclin A2 and CDK2/Cyclin E1 complexes, and more cells entered the subsequent cell cycle, leading to S phase arrest and mitotic catastrophe. We identified inhibition of CHK1 as a potential treatment for NSCLC and confirmed that inhibition of this kinase could overcome acquired gemcitabine resistance.

## GRAPHICAL ABSTRACT

**Figure:**
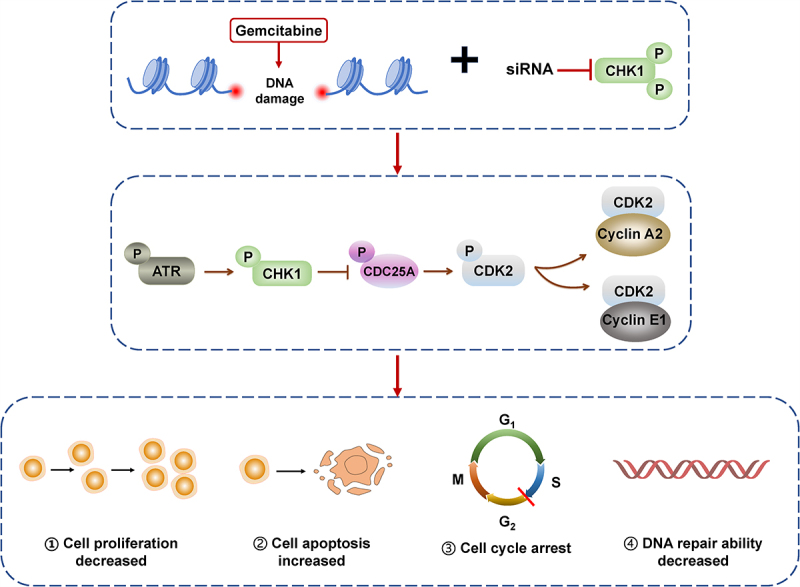

## Introduction

Lung cancer is the most common cancer in the world and the number one cause of cancer death, with non-small cell lung cancer (NSCLC) accounting for about 85% of lung cancers.^1^ According to estimates of global cancer incidence and mortality in 2022, lung cancer was the most common cancer (12.4% of all new cancer cases) and the leading cause of cancer deaths (18.7% of all cancer deaths) and return to the top one malignant tumor in incidence rate(15.3%) in 2022^2^. Compared with most countries, China has a relatively high death rate from lung cancer. Cao et al^3^ predicted that the death rate of lung cancer in China may increase by about 40% by 2030. Due to inadequate screening programs and the late onset of clinical symptoms, most patients with lung cancer are already in the middle and late stages when diagnosed. At this time, they have missed the best period of surgical treatment, and drug treatment is the last primary treatment method.^4^ However, acquired drug resistance seriously hinders the efficacy of treatment.^5^

Gemcitabine (Gem) is a pyrimidine nucleotide analog, which belongs to anti-metabolic anti-cancer drugs, and is also a specific anti-metabolic drug of the cell cycle. It mainly interferes with the process of the cycle by acting on the DNA synthesis stage of the cell cycle, namely the S phase, to prevent the self-replication of DNA. Under certain conditions, it can also block progression from the G_1_ phase to the S phase. Gemcitabine works by being transported into cells via a special nucleoside transporter. After entering the cell membrane via the nucleoside transporter, gemcitabine undergoes complex intracellular conversion to difluorodeoxycytidine diphosphate (dFdCDP) and difluorodeoxycytidine triphosphate (dFdCTP).^6^ DFdCDP is an effective inhibitor of ribonucleotide reductase (RR), which mainly consumes deoxyribonucleotide required for DNA synthesis by inhibiting the activity of RR, and reduces the amount of deoxycytidine diphosphate (dCDP) generated, thus inhibiting the synthesis of DNA.^7^ In addition, dFdCTP can bind to DNA, terminating the DNA strand when the extended strand binds to a nucleotide. Because the binding of dFdCTP to DNA seems to be resistant to common DNA repair mechanisms, this extra nucleotide may be an important reason for dFdCTP not to be detected by repair enzymes, resulting in DNA double-strand breaks and ultimately apoptosis.^8,9^ Despite the success of gemcitabine, its antitumor activity and toxicity vary from person to person.^10,11^ In addition, the development of drug resistance remains an important reason for the low response rate, poor efficacy, and severe toxicity of recurrent tumors.^12,13^

The correct operation of cell cycle depends on precise and orderly regulation of various proteins. The cell has an exact monitoring mechanism to ensure the fidelity of cell replication, which is called cell cycle checkpoint. When DNA damage or DNA replication is blocked, the cell cycle checkpoint is activated, and the operation of the cell cycle is stopped in time. The cell cycle cannot continue until DNA repair or troubleshooting. If DNA damage cannot be repaired, apoptosis is initiated. Cancer is a disease of cell cycle dysregulation. In the whole monitoring system of cell cycle progress, cell cycle checkpoints play a core role. Checkpoint kinase 1 (CHK1) and checkpoint kinase 2 (CHK2) are primary protein kinases in cell cycle checkpoint sites. They regulate downstream target protein activity and expression through signal transduction and lead to cell cycle arrest.^14,15^ Chemotherapeutic drug resistance is a major cause of tumor relapse, increased treatment difficulty, failure of therapy, and poor prognosis. And DNA damage response (DDR) is a leading cause to affect tumor chemotherapeutic drug resistance. In recent years, CHK1 has attracted extensive attention in regulating DDR from the whole globe. People expect to cause mitotic catastrophe and promote cell death, and CHK1 is a promising target for cancer therapy.^16^

CHK1 is a serine/threonine (Ser/Thr) protein kinase that controls the G_2_/M phase of DNA damage. The human CHK1 gene is located on chromosome 11q22–23 and is highly conserved in evolution. At the same time, CHK1 is also a significant regulator of DNA damage and replication detection sites, playing a critical role in regulating the cell cycle, participating in DNA damage repair, and influencing cell survival and apoptosis.^17^ Ataxia telangiectasia and Rad3-related kinase (ATR) activates and phosphorylates CHK1 in response to DNA damage caused by specific factors, thereby initiating the replication stress pathway and triggering the DNA damage repair system. This is called ATR-CHK1-Cell Division Cyclin 25A or cell division cyclin 25A (CDC25A) pathway.^18,19^ When ATR detects DNA double-strand breaks or long single-strand breaks, upstream ATR transmits DNA damage signals to CHK1 through mediators for activation.^20^ Activation of CHK1 inactivates CDC25A phosphatase, leading to cycle depended kinase (CDK) inactivation and the cell cycle arrest in S/G_2_ or G_2_/M phases.^21,22^ So far, high expression of CHK1 has been detected in multiple human tumors, such as breast cancer, lung cancer, liver cancer, gastric cancer, and nasopharyngeal cancer. Its expression is often positively correlated with tumor grade and disease recurrence.^23,24^ Some studies found that tumor cells with high expression of CHK1 were more resistant to DNA damage reactions caused by radiotherapy, chemotherapy, or other tumor treatments, which promoted the generation of tumor cells with a higher degree of malignancy and may also lead to frequent tumor recurrence and drug resistance.^25^

Therefore, we will explore the relationship between CHK1 and gemcitabine resistance through relevant experiments. And explain the reversal of gemcitabine resistance mediated by the ATR-CHK1-CDC25A pathway in this article.

## Results

### CHK1 is highly expressed in lung adenocarcinoma tissues

To clarify the correlation between CHK1 and tumor, and to understand the expression difference of CHK1 in tumor tissue and normal tissue, two databases were used for bioinformatics analysis in this study. TIMER 2.0 database showed that CHK1 was generally highly expressed in tumor tissues (Supplementary Figure S1 A). In the ENCORI database 526 lung adenocarcinoma tissues and 59 normal tissues were obtained. And the results showed that the expression of CHK1 in lung adenocarcinoma tissues increased by 5.17 times (*p* = 3.3e-40) (Supplementary Figure S1 B). Results from both databases showed that CHK1 overexpressed significantly in lung adenocarcinoma tissues and many other tumor tissues.

### siRNA effectively interferes with CHK1 expression and successfully determined gemcitabine-resistant A549 cells

Three small interfering RNA of CHK1 (siCHK1) sequences were designed to verify the specificity of the sequences. The siRNA was transfected into A549 cells to interfere with the expression of CHK1. And the siRNA negative control (siNC) group and siCHK1 group were set up to detect the mRNA and protein levels of CHK1 after transfection (Supplementary Figure S 2A, 2B, and 2C). Compared with the siNC group, the mRNA and protein levels of CHK1 in the three different siCHK1 sequences were significantly decreased after transfection, indicating that siRNA effectively interferes with the expression of CHK1 protein in cells. Combined with the results of mRNA level and protein level, the siCHK1–1 knockdown effect was the best, and siCHK1–1 was selected as the sequence fragment for all subsequent experiments.

Gemcitabine-resistant A549 cells (A549/G^+^) were generated by continuous administration of gemcitabine at low concentrations for 10 months. After cell culture for 24 h, sensitive cells and drug-resistant cells were cultured with a gemcitabine solutions of different concentration gradients for 72 h, and the cell survival rate under different concentrations of gemcitabine was calculated (Supplementary Figure S2D). The results showed that the cell survival rate of the sensitive group was lower than that of the drug-resistant group at the same concentration except for 5 μmol/L. At the same time, the IC50 value of the sensitive group was lower than that of the resistant group [2.834 μM (95% CI: 0.561 μM~6.476 μM) vs 33.582 μM(95% CI: 13.776 μM~74.051 μM)]. All of these indicate that the A549 cell line that induced gemcitabine tolerance before is still resistant. And it can be used for further experimental studies.

### CHK1 inhibition combined with gemcitabine reduced cell proliferation ability

**Figure 1.**
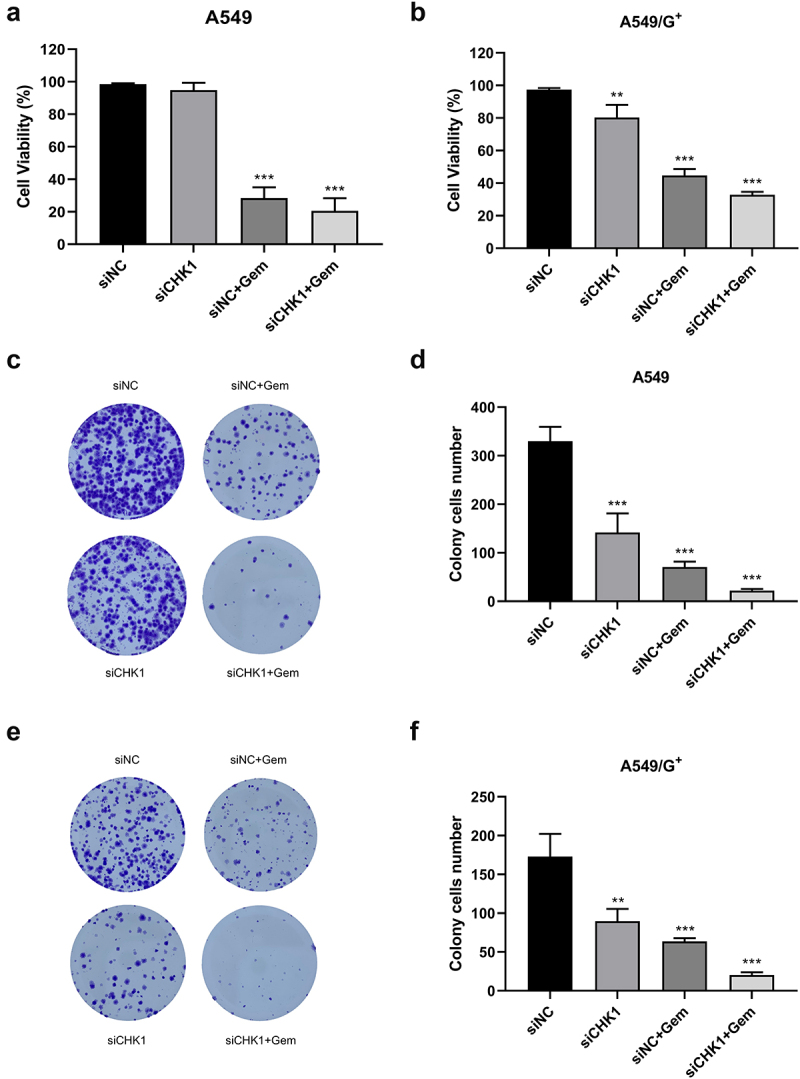
CHK1 inhibition combined with gemcitabine reduced proliferation ability of A549 and A549/G^+^ cells.

We initially examined whether siCHK1 enhances the cytotoxic effects of gemcitabine in A549 and A549/G^+^ cells. The CCK-8 assay revealed that relative cell viability was lower in the two cell lines treated with the combination of gemcitabine and siCHK1 than in those treated with each one alone (Figure 1a,b). Our results demonstrate that CHK1 knockdown by siCHK1 in A549 cells did not show significant differences in proliferation rates compared to the siNC control group. However, in drug-resistant A549/G^+^ cells, treatment with the same concentration of siCHK1 exhibited more pronounced effects, suggesting that the resistant cells may be more sensitive to CHK1 depletion. Similarly, combined treatment with CHK1 inhibition and gemcitabine suppressed the colony formation of two cells (Figure 1c–f). The results of the clone formation experiment were consistent with those of the CCK-8 assay, that is, the proliferation ability of A549 cells and A549/G^+^ cells could be reduced obviously by combination treatment.

Cells were treated with 10 nM siNC, 10 nM siCHK1, 10 nM siNC plus 10 µM Gemcitabine (siNC+Gem), or 10 nM siCHK1 plus 10 µM Gemcitabine (siCHK1+Gem). a and b: Bar graphs showing comparison of cell survival rate among groups of A549 cells and A549/G^+^ cells, respectively. Cells were treated at the indicated doses for 72 h and data are presented as the mean ± SD of three independent experiment. c and e: Cell clonal formation of each group in A549 cells and A549/G^+^ cells, respectively. d and f: Bar graphs showing colony cells number of each group in A549 cells and A549/G^+^ cells, respectively. Cells were harvested after 14 days and data are presented as the mean ± SD of three independent experiment. Compared with the siNC group, ***: *p* < .001, **: *p* < .01.

We also found that A549 cells grew faster and were more sensitive to gemcitabine at the same concentration. As can be seen from the clone formation experiment, the density of A549 cells in the siNC group was higher than A549/G^+^ cells. And the colony cell number of A549 cells was reduced significantly in the siNC + Gemcitabine (siNC+Gem) group after treatment. Meanwhile, A549/G^+^ cells had bigger volume and stronger adhesion ability than A549 cells, which was consistent with the characteristics of drug-resistant cells.

### CHK1 inhibition combined with gemcitabine induce cell apoptosis

**Figure 2.**
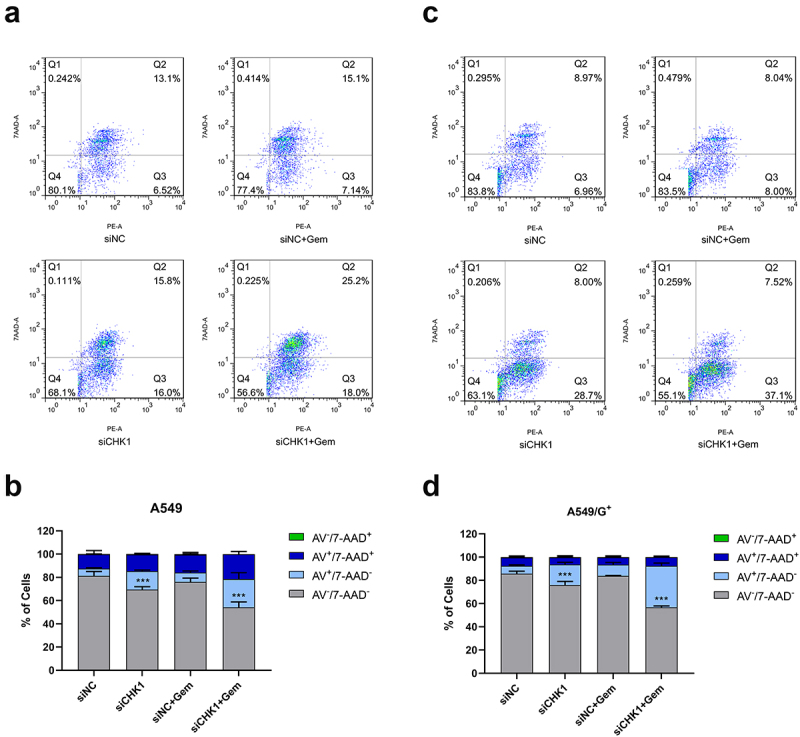
CHK1 inhibition combined with gemcitabine promote cell death in A549 and A549/G^+^ cells.

To evaluate the potential induction of cell apoptosis, we treated the two cell lines with the reagents alone or in combination with the CHK1 inhibition. And after 48 h of treatment, exposed to an Annexin V-phycoerythrin/7-amino-actinomycin D (AV/7-AAD) solution to allow cell apoptosis determination by flow cytometry. In the bivariate flow cytometry scatter plots (Figure 2a,c), the upper right quadrant stands for the late apoptotic cells (AV^+^/7-AAD^+^ cell population); the lower right quadrant signifies early apoptotic cells (AV^+^/7-AAD^−^ cell population); the lower left quadrant stands for living cells (AV^−^/7-AAD^−^ cell population); the upper left quadrant stands for the mechanical injury cells (AV^−^/7-AAD^+^ cell population). The results showed that the total apoptosis rate (early apoptosis rate + late apoptosis rate) increased significantly in both groups cells after combined treatment. In addition, after CHK1 inhibition and gemcitabine treatment, both early and late apoptotic cell populations of A549 cells were significantly increased, and the number of viable cells decreased significantly. However, in A549/G^+^ cells, the number of early apoptotic cells increased, and that of late apoptotic cells did not change (Figure 2b,d). We believed that under the same treatment conditions, A549/G^+^ cells were partially resistant to apoptosis after combined treatment, and inhibition of CHK1 alone also significantly changed the apoptosis rate of the two cell lines. However, we also noticed that there was no difference in apoptosis between the siNC+Gem group and the siNC group in the two cell lines. This is primarily because Gem, when administered at low doses or for short-term treatment, preferentially inhibits cell proliferation through DNA synthesis blockade and replication stress, rather than directly activating the apoptotic pathways. This observation is consistent with our CCK8 and colony formation assay results, which demonstrated that the siNC+Gem group exhibited significantly reduced proliferation capacity without a marked increase in apoptosis.

Cells were seeded and then exposed at the same doses of gemcitabine indicated in Figure 1 for 48 h. The percentages of AV ^±^ and 7AAD ^±^ cells were determined by flow cytometry. a and c: 2D scatter plots showing the apoptosis rate of A549 cells and A549/G^+^. b and d: Bar graphs showing the number of cells in different states for A549 and A549/G^+^ cells, including mechanical injury (Green), late apoptosis (Dark blue), early apoptosis (light blue), and living cells (Gray). Data are presented as the mean ± SD deviation of triplicate experiments. Compared with the siNC group, ***: *P* < 0.001.

**Figure 3.**
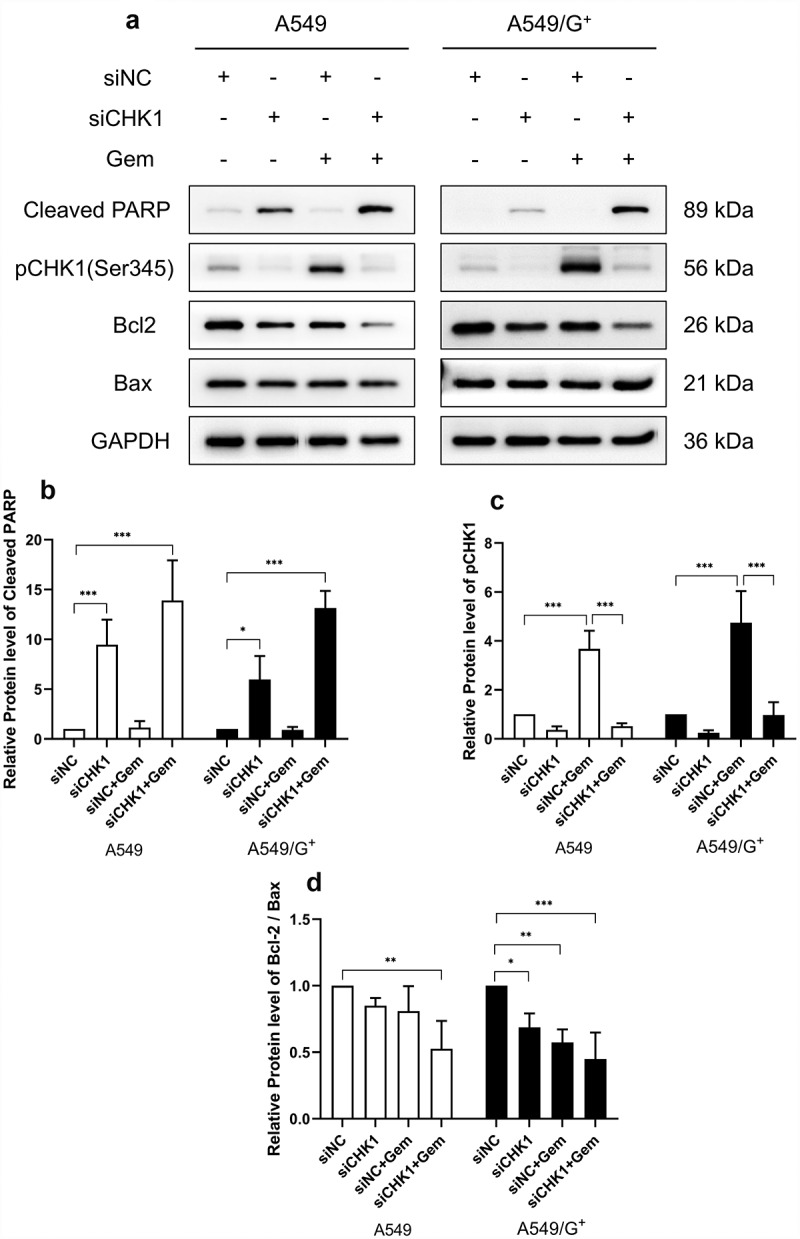
The expression of apoptotic proteins in A549 and A549/G^+^ cells after combined treatment.

Subsequently, the expression levels of apoptosis-related proteins cleaved poly (ADP-ribose)-polymerase (Cleaved PARP), Bcl-2, and Bax were detected by Western blot. Cleaved PARP is an indicator of apoptosis. Results showed that compared with the siNC group, Cleaved PARP in the siCHK1 group and siCHK1+Gem group were increased, indicating an increased cell apoptosis rate. Bcl-2 was an anti-apoptosis protein, and both cells showed low expression of Bcl-2 after combined treatment. Meanwhile, the expression of pro-apoptotic indicator Bax did not change. Using the Bcl-2/Bax ratio to indicate the overall apoptosis level, the apoptosis degree of the siCHK1+Gem group of both cells increased significantly, indicating an increased apoptosis rate, which was consistent with the results of the flow cytometry apoptosis experiment (Figure 3).

a:The expression levels of apoptosis markers Cleaved PARP, Bcl-2, Bax, and pCHK1(Ser345) were detected by Western blotting. b, and c: Bar graphs of relative expression level of Cleaved PARP, and pCHK1(Ser345) respectively. d: Bar graphs of relative expression ratio of Bcl-2/Bax. Glyceraldehyde-3-phosphate dehydrogenase(GAPDH) was used as a loading control. Compared with the siNC group, ***: *P*< 0.001; **: *P*< 0.01; *: *P*< 0.05.

CHK1 inhibition combined with gemcitabine induced DNA damage response and affected homologous-recombination repair ability

**Figure 4.**
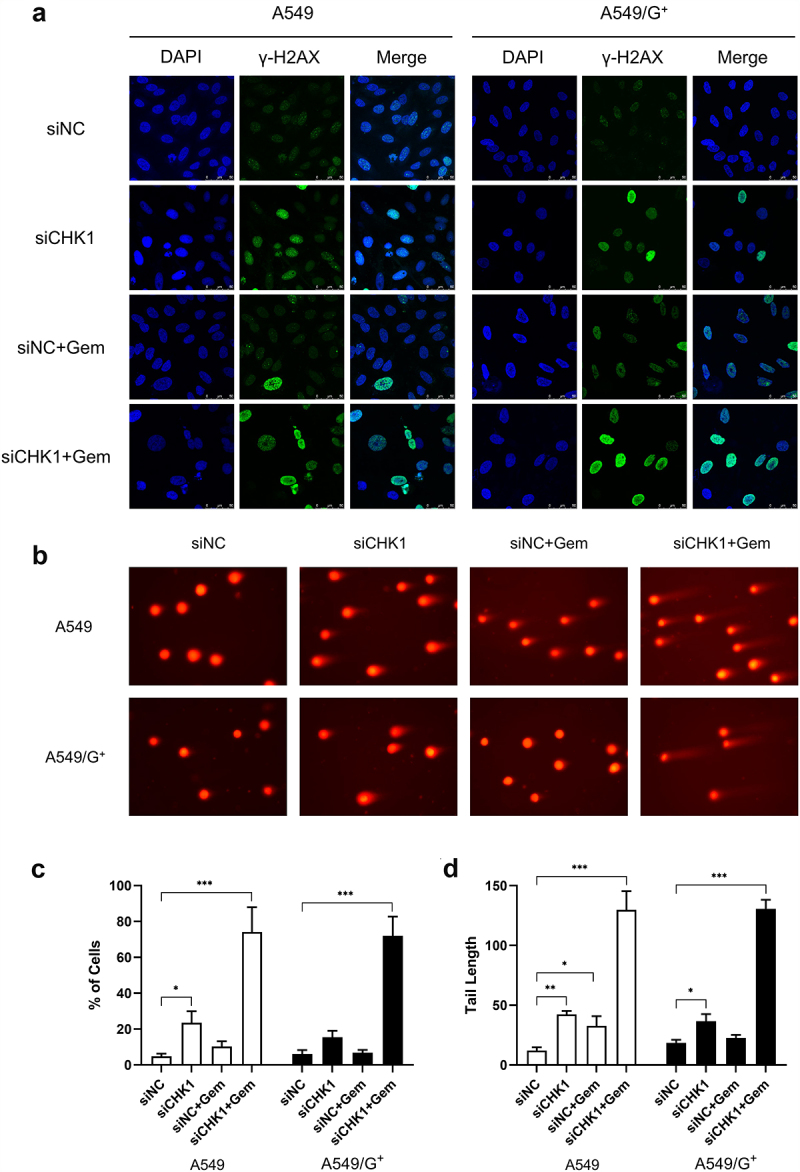
CHK1 inhibition and gemcitabine treatment induces DNA double-strand break.

We have observed the degree of DNA double strand breaks caused by combined treatment in the same group of A549 and A549/G^+^ by immunofluorescence test and neutral comet assay. γ-H2AX is a marker of DNA double-strand fracture damage. Immunofluorescence results showed that the siCHK1+Gem group produced a large proportion of γ-H2AX green fluorescence. And most of the green fluorescence is around the periphery of the nucleus. It was not the focalization of γ-H2AX but the pan-nucleation of γ-H2AX, and it was the result of more DNA damage and DNA replication stress caused by the combined treatment. In the A549 cell line, compared with the siNC group, the siCHK1 and siCHK1+Gem groups showed different degrees of cell morphology changes, such as nuclear vacuolation, and apoptotic bodies. But A549/G^+^ cells did not obviously show such morphological change. All of these indicated that the combined treatment had a more intense damage effect on sensitive cell strains (Figure 4a,c).

a: Immunocytochemistry demonstrated that pan-nuclear γ-H2AX was significantly increased by the combination of CHK1 inhibition and gemcitabine. Cells were stained with anti-phospho-histone H2AX-Ser139 (green) and 4,6-diamidino-2-phenylindole (DAPI, nuclear DNA, blue). The scale bar was shown at 50 µm and the cells containing more than 5 γ-H2AX foci were counted under blinded conditions. b: Neutral comet experiment demonstrated that both siCHK1+Gem groups produced a long comet trailing. DNA double-strand breaks were evaluated by the comet tail length. Data represent mean ± SD deviation of triplicate experiments. c: Bar graphs showing the number of cells with pan-nuclear γ-H2AX in Figure 6a. d:Bar graphs show the percentage of comet tail length analyzed in at least 100 cells each slide. Compared with the siNC group, ***: *P*< 0.001; **: *P*< 0.01; *: *P*< 0.05.

The neutral comet experiment can effectively detect and quantitatively analyze the degree of DNA damage in cells. The more serious the damage is the more DNA chain breaks. Under the same electrophoresis conditions, the longer the distance of cell migration and the longer the tail length are. The results showed that both siCHK1+Gem groups produced a long comet trailing phenomenon (Figures 4b,d).

**Figure 5.**
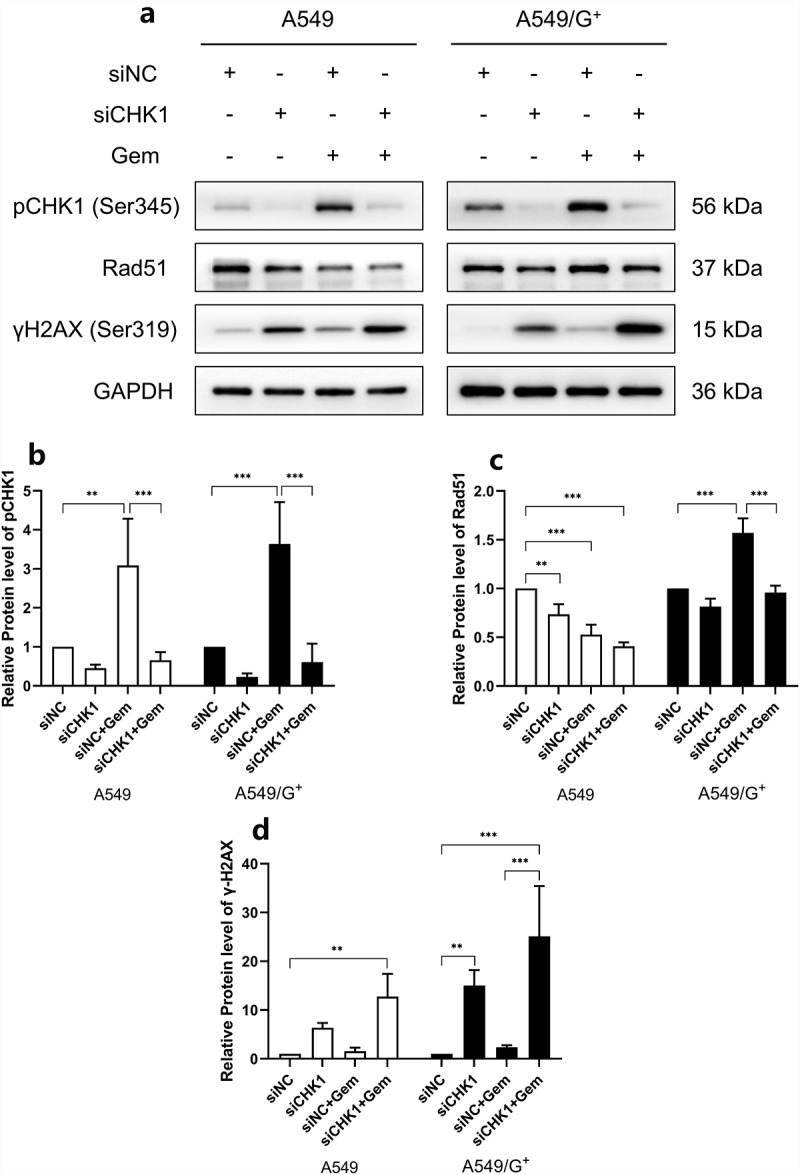
The expression of damage repair proteins in A549 and A549/G^+^ cells after combined treatment.

Subsequently, we detect the expression levels of γ-H2AX and Rad51 with a Western blot assay. The results showed that compared with the siNC group, the expression level of γ-H2AX in the siCHK1 group and the siCHK1+Gem group was significantly increased, indicating that the combined treatment increased the accumulation of γ-H2AX produced by DNA damage. A549 cells damaged by gemcitabine treatment alone, and the expression of γ-H2AX increased, but it did not affect A549/G^+^. The pCHK1 was activated and its expression increased under the action of drugs. Rad51 is a key protein in the homologous-recombination repair (HR) pathway. The stronger the HR ability, the higher the expression level of Rad51. The result showed that the expression of Rad51 in A549 cells was decreased after CHK1 inhibition, indicating that medication resulted in a relatively reduced repair ability. While the expression level of Rad51 was enhanced in A549/G^+^ cells after gemcitabine treatment, indicating that drug-resistant cells had better repair ability than sensitive cells. The combined treatment reduced the expression of Rad51 in A549 cells. For A549/G^+^ cells, the expression level of Rad51 in the siCHK1+Gem group was lower than that in the siNC+Gem group, indicating that the combined treatment reduced the activity of HR ability was relatively reduced. At this time, the repair ability could not completely repair the cell damage, resulting in cell death (Figure 5).

a:The expression levels of pCHK1 (Ser345), Rad51, and γH2AX (Ser139) were evaluated by Western blot. b and c: Bar graphs showing relative expression level of pCHK1 (Ser345), and Rad51, respectively. d: Bar graph of relative expression level ofγH2AX (Ser139). GAPDH was used as a loading control. Compared with the siNC group, ***: *P*< 0.001; **: *P*< 0.01; *: *P*< 0.05.

### CHK1 inhibition combined with gemcitabine resulted in S phase arrest and RRM2 down regulation

Cell cycle experiments were performed on A549 and A549/G^+^ cells in the same group to observe the cell cycle changes caused by CHK1 inhibition combined with gemcitabine. The results showed that gemcitabine increased the number of cells in the G_1_ phase and decreased the number of cells in the S phase in the siNC+Gem in A549 cells, indicating that gemcitabine can block A549 cells in the G_1_ phase. As for A549/G^+^ cells, the number of cells in the S phase increased in the siNC+Gem group under gemcitabine treatment. These indicated that the drug could block A549/G^+^ cells at the S phase, which also manifested that A549/G^+^ could partially tolerate gemcitabine treatment, making the cell cycle passed the G_1_ phase.

**Figure 6.**
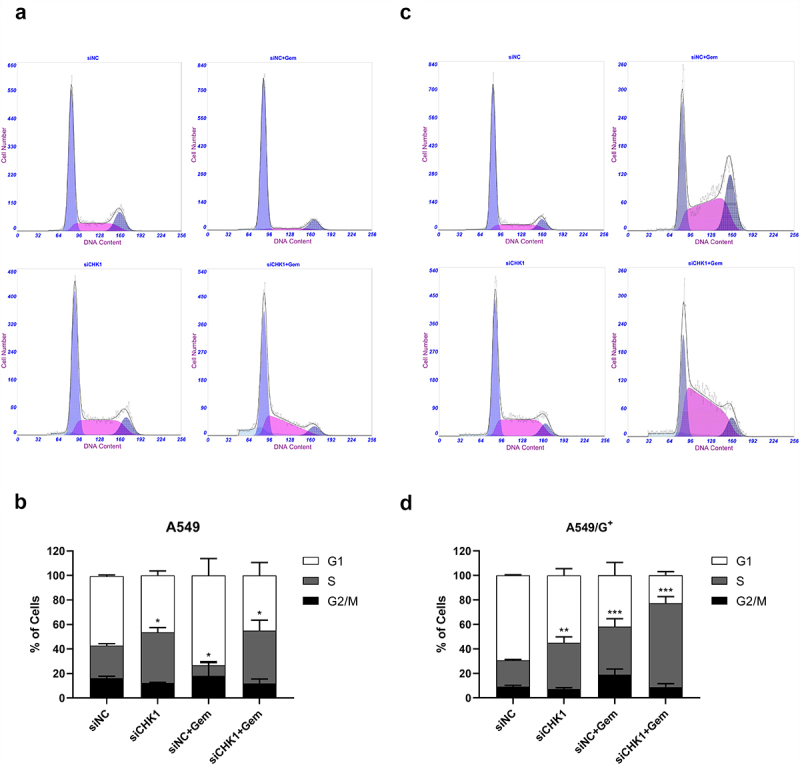
CHK1 inhibition and gemcitabine treatment induced S phase arrest in cell cycle.

Compared with the siNC group, the siCHK1 group had more S phase cells in A549. The S phase cell number of the siNC+Gem group decreased after drug treatment. But the siCHK1+Gem group could partially recover the reduced S phase, increasing the S phase cell number, which demonstrated that more A549 cells could pass the G_1_ phase and reach the S phase after combined treatment (Figure 6a,b). The same phenomenon also appeared in A549/G^+^ cells, siCHK1 group increased the number of S phase cells, siCHK1+Gem group further significantly increased the proportion of S phase cells. All of this indicated that after combined treatment, drug-resistant cells might be more likely than sensitive cells to pass the G_1_ phase and finally be blocked in the S phase (Figure 6c,d). Damaged cells are not repaired and enter the abnormal cell cycle, resulting in mitosis disaster.

Cells were exposed to the indicated treatment for 24 h. After being harvested and fixed, cells were stained with propidium iodide (PI) to assess the cell cycle distribution. Upper panels show the PI histograms; lower panels show the proportion of cells in each phase of the cell cycle after the indicated treatments. a and c: Cell cycle histograms of A549 cells and A549/G+ cells, respectively. b and d: Bar graphs showing the percentage of cells in each phase of the cell cycle for A549 cells and A549/G^+^ cells, respectively. Data represent mean ± SD deviation of triplicate experiments. Compared with the siNC group, ***: *P*< 0.001, **: *P*< 0.01, *: *P*< 0.05.

**Figure 7.**
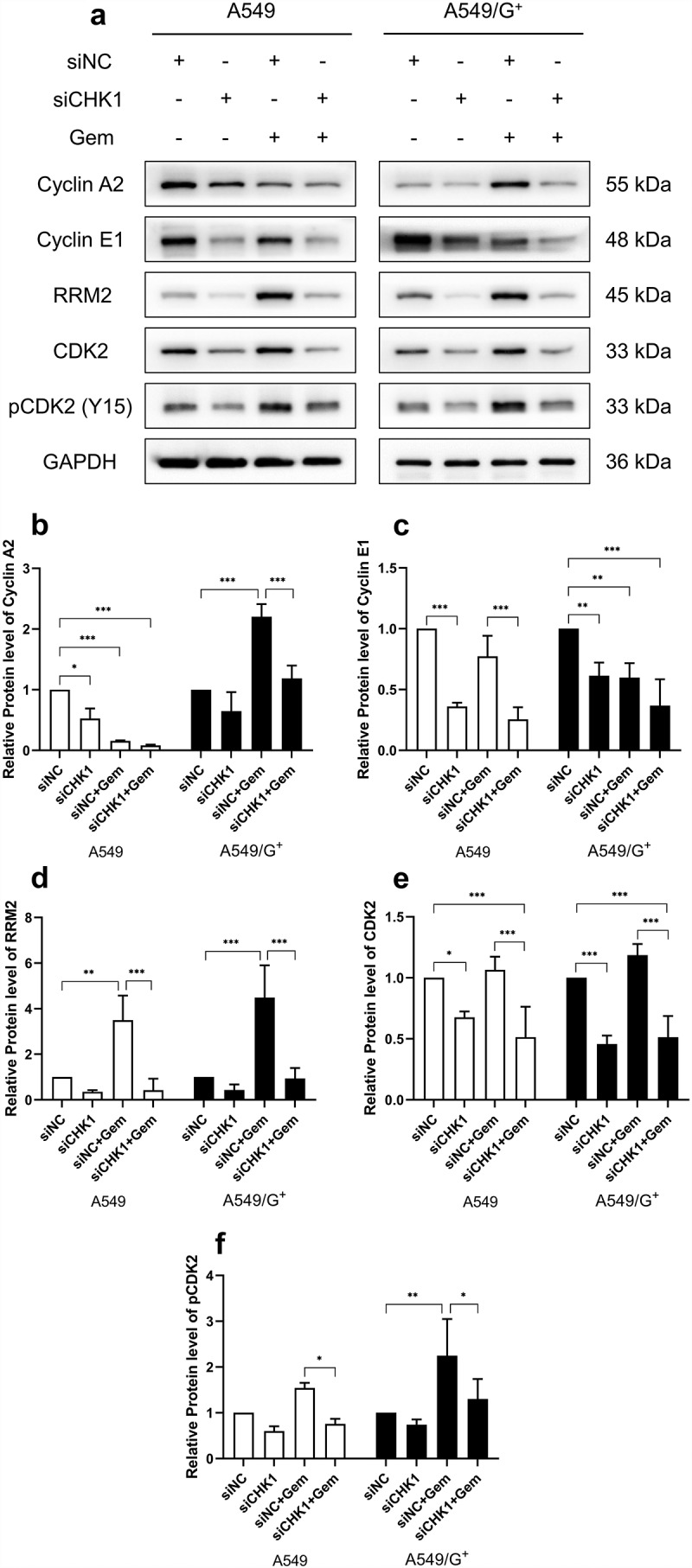
The expression of cell cycle related proteins in A549 and A549/G^+^ cells after combined treatment.

Then, we used Western blot to detect the expression levels of cell cycle-related proteins: Cyclin A2, Cyclin E1, RRM2, CDK2, and pCDK2. The binding of CDK2 to Cyclin A2 is a necessary condition for initiating and progressing G_2_ phase events. Meanwhile, CDK2 needs to bind Cyclin E1 to trigger the S phase. When CDK2 dephosphorylation is activated, the level of its phosphorylated form, pCDK2 (Y15), decreases. The results showed that the expression levels of free CDK2, Cyclin A2, and Cyclin E1 decreased after CHK1 knockdown treatment in both cell lines regardless of whether gemcitabine was added or not. This indicated the binding between CDK2/Cyclin A2 and CDK2/Cyclin E1 was increasing. These complexes can induce cells to enter the subsequent S and G_2_ phases. The expression of pCDK2 (Y15) decreased after activation, showing that CDK2 further completed the binding with Cyclin A2 and E1. As shown in Figure 7, under the action of gemcitabine, RRM2 was activated, and then its expression increased, while siCHK1+Gem treatment reduced RRM2. The results indicated that the combined treatment made cells sensitized to gemcitabine and promoted cell death. In short, it helped cells overcome resistance to gemcitabine.

a:The expression levels of Cyclin A2, Cyclin E1, RRM2, CDK2, and pCDK2 (Y15) were evaluated by Western blot. b, c, d, e, and f: Bar graphs showing the relative expression levels of Cyclin A2, Cyclin E1, RRM2, CDK2, and pCDK2 (Y15), respectively. GAPDH was used as a loading control; Compared with siNC group, ***: *P*< 0.001; **: *P*< 0.01; *: *P*< 0.05.

### CHK1 inhibition combined with gemcitabine affects the ATR-CHK1-CDC25A pathway to overcome gemcitabine resistance

**Figure 8.**
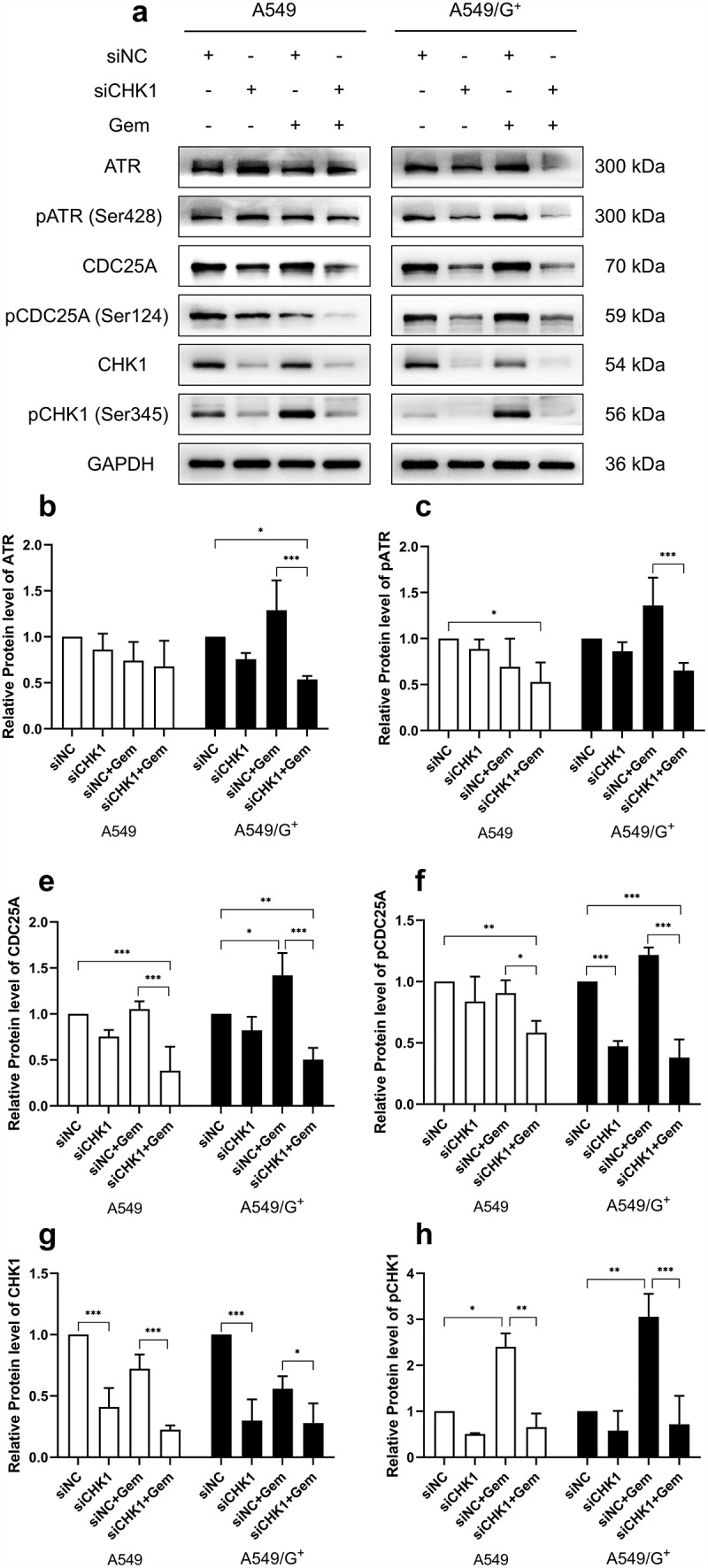
The expression of ATR-CHK1-CDC25A pathway protein in A549 and A549/G^+^ cells after combined treatment.

By reviewing the literature on cellular replication stress and upstream and downstream pathways related to CHK1 protein, Western blot was used to detect the expression of ATR-CHK1-CDC25A pathway-related proteins ATR, pATR (Ser428), CHK1, pCHK1 (Ser345), CDC25A and pCDC25A (Ser124) in each group of cells. The results showed that there was no significant change in protein levels of ATR and pATR in each group of A549 cells, but ATR and pATR were all activated by drugs in A549/G^+^ cells. The expression levels of CHK1 and pCHK1 decreased significantly after knockout treatment. Gemcitabine activated pCHK1 and made its expression level increased. According to relevant literature, pCHK1 inhibits CDC25A through phosphorylation. CDC25A, as a dephosphorylated protein, decreases its phosphorylation state when activated and pCDC25A expressed significantly reduced after CHK1 knockdown (Figure 8).

a:The expression levels of ATR, pATR (Ser428), CDC25A, pCDC25A (Ser124), CHK1, and pCHK1 (Ser345) were evaluated by Western blot. b, c, d, e, f, g, and h: Bar graphs of relative expression level of ATR, pATR(Ser428), CDC25A, pCDC25A (Ser124), CHK1, pCHK1 (Ser345), respectively. GAPDH was used as a loading control. Compared with siNC group, ***: *p* < .001; **: *p* < .01; *: *p* < .05.

When drugs damage cells, the ATR-CHK1-CDC25A pathway will be activated. Under normal regulation, ATR-CHK1 regulates CDC25A negatively, so CDC25A will not be activated, and neither will its downstream CDK2. That is, CDK2 cannot bind to Cyclin A2 and Cyclin E1. The CDK2/Cyclin A2 and CDK2/Cyclin E1 complexes could not be formed between the two cells, leading to the failure of cell cycle progression, and the two cell lines were blocked, with A549 cells blocked in the G_1_ phase and A549/G^+^ cell blocked in S phase. After siRNA inhibited CHK1‘s expression, CDC25A was activated to release the phosphorylated group. And CDK2 was activated and combined with Cyclin A2 and Cyclin E1, resulting in the damaged cells crossing the monitoring point of cell cycle detection and entering the subsequent cell cycle. These could cause mitotic disaster finally. Thus, resistant cells A549/G^+^ can achieve the effect of overcoming gemcitabine resistance.

## Discussion

NSCLC is one of the most common cancers worldwide, with a high mortality rate.^2^ As a first-line chemotherapy drug for treating NSCLC, the efficacy of gemcitabine is severely limited by drug resistance.^26,27^ In this study, we investigated the potential of combining CHK1 inhibitors with gemcitabine in overcoming NSCLC drug resistance.

The occurrence of drug resistance is the result of multiple factors and mechanisms. The formation of gemcitabine resistance in NSCLC may involve multiple mechanisms, including mutations in drug targets, upregulation of drug-metabolizing enzymes, enhanced DNA repair ability, and changes in cell cycle regulation.^28^ Among them, CHK1, as a key cell cycle checkpoint kinase, is crucial in maintaining genomic stability and promoting DNA damage repair.^29^ Our research results indicate that inhibition of CHK1 can enhance the killing effect of gemcitabine on drug-resistant NSCLC cell lines.

Our experimental data show that the combination of CHK1 inhibition and gemcitabine can significantly reduce the proliferation ability of drug-resistant cell lines, and enhance the efficacy of gemcitabine by promoting cell apoptosis and DNA damage response. In addition, we also observed that combination therapy could lead to S-phase blockade and activation of the ATR-CHK1-CDC25A signaling pathway, which may be one of the molecular mechanisms for overcoming drug resistance. These findings are consistent with previous researches that show that the inhibition of CHK1 can increase the activity of various DNA damage or DNA synthesis inhibition therapies in different cancer cells.^30–35^ As is well known, the DNA Damage Response (DDR) signaling pathway includes the ATM/ATR-CHK1/CHK2-Cdc25s pathway (for rapid, reversible reactions) and the p53-dependent pathway (for slower, irreversible reactions).^36^ P53 is a central downstream checkpoint signaling protein responsible for apoptosis response. The ATR-CHK1 signaling pathway is crucial for inhibiting caspase-3-dependent apoptosis response after replication stress.^37^ In addition, CHK1 also blocks Caspase-2-dependent apoptotic responses independently of P53, Bcl-2, and Caspase-3. This increases the possibility of an unknown link between CHK1 and apoptotic signaling transduction.^38^ Therefore, for the study of CHK1 on gemcitabine resistance in A549 cells in this article, it cannot be ruled out that there is a phenomenon of a P53-dependent pathway leading to cell apoptosis.

In recent years, CHK1 inhibitors have been studied in clinical trials. CHK1 inhibitors have potential advantages in treating NSCLC: (1) Enhanced efficacy: CHK1 inhibitors can enhance the killing effect of gemcitabine on drug-resistant cells, providing a new strategy for treating NSCLC. (2) Overcoming drug resistance: By inhibiting CHK1, the phenotype of drug-resistant cells can be reversed, restoring drug sensitivity. (3) Potential synergistic effects: Combining CHK1 inhibitors with other treatment modalities (such as radiotherapy, and other chemotherapy drugs) may have synergistic effects, providing more treatment options. (4) Molecular targeted therapy: CHK1 inhibitors, as a therapical targeted molecular, may provide more precise and personalized treatment methods.

Although CHK1 inhibitors have shown positive effects in many studies,^39–41^ their specificity and potential off-target effects in vivo are currently unclear. In addition, inhibiting CHK1 may affect the function of other cell cycle checkpoint proteins, which may cause unexpected side effects. To overcome these limitations, future research can evaluate the effectiveness of CHK1 inhibitors in more NSCLC cell lines and clinical samples. Evaluate the safety and efficacy of drugs through in vivo model studies. In addition, exploring the combination of CHK1 inhibitors with other treatment methods to improve treatment efficacy. Study other mechanisms of drug resistance formation and develop new therapeutic targets. Finally, clinical trials will be conducted to validate the potential of CHK1 inhibitors in the treatment of NSCLC.

There may be some limitations in the present research. We only conducted experiments on A549 and its derived drug-resistant cell lines, making it difficult to generalize these findings directly to all NSCLC patients. In addition, we have not yet explored other DNA repair pathways activated after CHK1 inhibition. Future research will focus on the following aspects:

Firstly, expanding the sample range to include more NSCLC cell lines and clinical samples.

Secondly, exploring the combined use of CHK1 inhibitors with other drugs.

Thirdly, identifying molecular markers of drug resistance to guide the development of personalized treatment strategies.

In summary, this study provides experimental evidence for the combined treatment of NSCLC with CHK1 inhibition and gemcitabine and offers a new perspective for future drug resistance research. We look forward to CHK1 inhibition becoming a powerful tool to overcome gemcitabine resistance in NSCLC.

## Conclusions

Our results found that CHK1 inhibition combined with gemcitabine had a better anti-tumor effect on A549 cells. Furthermore, our study demonstrates the promise of drugs that target the detection of checkpoint kinase and DNA replication stress responses as therapeutic strategies for lung cancer.

Therefore, we support the development of this approach for clinical studies of CHK1 inhibitors in combination with gemcitabine in patients with advanced lung cancer.

## Materials and methods

### Cell lines, culture conditions, and reagent

The NSCLC cell line A549 was purchased from the Institute of Oncology in Peking Union Medical College and sourced from the American Type Culture Collection (ATCC). Gemcitabine-resistant A549 cells (A549/G^+^) were established in 2019 by our research group and has been preserved to this day. The specific process can be found in our previous research.^42^ In short, A549 cells were cultured using a low concentration (0.1 μM) continuous dosing method until they could grow in a high concentration (3 μM) gemcitabine medium. Then, the cells were continuously passaged in a medium containing 3 μM gemcitabine. The induction process lasted for 39 weeks. Finally, verify that its resistance index reaches 3 or above, which can be used for experiments. The A549 cells and A549/G^+^ cells were grown in Dulbecco’s modified Eagle’s (DMEM; Hyclone) and the media was supplemented with 10% fetal bovine serum (FBS; Sangon Biotech, China), 100 U/mL penicillin and 100 μg/mL streptomycin (Solarbio, China) at 37°C in a 5% CO_2_ atmosphere. To passage the cell lines, each confluent monolayer was washed with phosphate-buffered saline (PBS; Solarbio, China) and detached with a 0.05% trypsin/0.02% ethylenediaminetetraacetic acid (EDTA) solution (Gibco, USA). Gemcitabine hydrochloride was purchased from Eli Lilly and Company (USA) and prepared in water. To knock down the expression of target genes, cell lines were transfected with siRNA (10 nM). The siRNAs were designed and synthesized by Guangzhou Ruibo Biotechnology Co., Ltd (Ruibo, China). Their sequences were as follows: siCHK1–1, GTGATGGATTGGAGTTCAA; siCHK1–2, GAAAGAGATCTGTATCAAT; siCHK1–3, CAGTGAAGATTGTAGATAT. All siRNAs are synthesized using chemical synthesis methods.

The specific transfection process is as follows: 3 × 10^5^ cells were plated one day before transfection in six-well plates. Cells were transfected with siRNA specific to Chk1 (Ribobio, China), or scrambled siRNA (Ribobio, China) using Lipofectamine® 3000 Transfection Kit (Thermo Fisher Scientific, USA) for 24 h, followed by treatment with gemcitabine.

### Reverse transcription-quantitative polymerase chain reaction (rt-qPCR) assay

Total RNA was isolated from cells with RNAiso Plus (Takara, Japan) and cDNA was produced by the Prime Script RT Master Mix (Takara, Japan). The TB Green Premix Ex Taq™ II Kit (Takara, Japan) was used to detect relative mRNA expression by real-time quantitative PCR instrument (Thermo Fisher Scientific, USA) with 40 cycles of PCR thermocycling. The human CHK1 and GAPDH primer sequences were designed and synthesized by Shanghai Sangon Biotechnology Co., Ltd (Sangon Biotech, China). The primer sequences were as follows: GAPDH forward primer sequences, 5’-CAG GAG GCA TTG CTG ATG AT −3’ and reverse primer sequences, 5’-GAA GGC TGG GGC TCA TTT-3’; CHK1 forward primer sequences, 5’-TTC AGG TGG TGT GTC AGA GTC TCC-3’ and reverse primer sequences, 5’-TGT GCG GGG TTC TGG CTG AG-3’. The mRNA expression of the target gene was calculated by the 2^−ΔΔT^ method and normalized against the expression level of GAPDH.

### Western Blot Analysis

Protein samples were prepared by lysing cells in radioimmunoprecipitation assay (RIPA) buffer (Beyotime, China) containing a protease inhibitor (Applygen, China) and a phosphatase inhibitor cocktail (Beyotime, China) and were stored at 4°C for 30 min and vortexed every 5 minutes. Lysates were centrifuged to obtain protein extracts for western blotting. The BCA (bicinchoninic acid, Thermo Fisher Scientific, USA) method was used to detect the protein concentration. SDS-PAGE (sodium dodecyl sulfate-polyacrylamide gel electrophoresis) (10% gel, Beyotime, China) were used for protein electrophoresis (equal amount per protein sample), and the separated proteins were transferred to PVDF (polyvinylidene fluoride) membranes (Millipore, USA). The membranes were blocked with 5% skimmed milk in TBS (tris buffered saline) with 0.2% Tween-20 and incubated with the following primary antibodies at 4°C overnight: GAPDH (diluted 1:1000, Beyotime, cat. no. AF1186, China), Cleaved PARP (poly ADP-ribose polymerase, diluted 1:1000, CST, cat. no. 5625, USA), Bcl-2 (B-cell lymphoma-2, diluted 1:1000, Bioworld, cat. no. BS70205, USA), Bax (BCL2-associated X protein, diluted 1:10000, Abcam, cat. no. ab32503, USA), phospho-Chk1-Ser345 (diluted 1:1000, CST, cat. no. 2348), CHK1 (diluted 1:10000, Abcam, cat. no. ab40866, USA), Rad51 (radiation-sensitive 51, diluted 1:1000, Abcam, cat. no. ab133534, USA), phospho-Histone H2AX (H2A histone family member X)-Ser139 (diluted 1:1000, CST, cat. no. 9718, USA), Cyclin A2 (diluted 1:2000, CST, cat. no. 4656, USA), Cyclin E1 (diluted 1:1000, CST, cat. no. 4129, USA), RRM2 (RR Regulatory Subunit M2, diluted 1:10000, Abcam, cat. no. ab172476, USA), CDK2 (diluted 1:1000, CST, cat. no. 2546, USA), phospho-CDK2-Y15 (diluted 1:10000, Abcam, cat. no. ab76146, USA), ATR (diluted 1:1000, CST, cat. no. 13934, USA), phospho-ATR-Ser428 (diluted 1:1000, CST, cat. no. 2853, USA), CDC25A (diluted 1:1000, CST, cat. no. 3652, USA), phospho-Cdc25A-Ser124 (diluted 1:1000, Abcam, cat. no. ab156574, USA). On the next day, the membranes were washed and then incubated for 2 hours at room temperature with HRP (horseradish peroxidase)-conjugated anti-rabbit or anti-mouse secondary antibodies at the dilution of 1:1000 (cat. no. A0208 and A0216; both from Beyotime, China). Labeled proteins were detected and taken photos using an enhanced chemiluminescence detection system (Azure Biosystems, USA). The band quantification was conducted by Image J 1.51K software (National Institute Health(NIH), USA) and normalized against GAPDH level. The above experiment was conducted three times in total.

### CCK-8 cytotoxicity experiments

Cell counting kit-8 (CCK-8, Dojindo Laboratories, Japan) cytotoxicity experiments were used to detect the dose-response and drug combination studies. Briefly, A549 and A549/G^+^ cells (or cells after the transfection of siNC (siRNA negative control) and siCHK1) were seeded at 3000 cells/well in 96-well plates and cultured overnight. Cells were incubated with various concentrations of gemcitabine (0, 5, 10, 50, 100, and 500 μM) for 72 h. Then, each well was added 10 μl of CCK-8 reagent and the plates were incubated for 1 hour at 37°C in the dark. The absorbance at 450 nm was detected by the iMark microplate reader (Bio-Rad, USA). The Probit regression model and calculate its IC50 (half maximal inhibitory concentration) value with Statistical Product and Service Solutions (SPSS, International Business Machines Corporation, USA)25.0 software.

### Colony formation experiments

Cells after the transfection of siNC and siCHK1 were seeded in 6-well plates at a density of 800 cells/well, allowed to attach for 24 h, and then treated with 0 or 10 μM gemcitabine for 24 h. The medium was changed in the middle term of treatment to prevent the drying and evaporation of the culture medium during long-term culture. After 14 days, stop culture and gray-white clonal spots can be seen. Cells were fixed and stained with crystal violet solution. The colonies were visualized and counted with Image J 1.51K software (NIH, USA).

### Cell cycle assay

After the transfection, the cells were seeded in 6-well plates at a density of 2 × 10^5^ cells/well, allowed to attach for 24 h, and then treated with 0 or 10 μM gemcitabine for 24 h. Then, cells were harvested by trypsinization, washed with cold PBS, and fixed with 70% ethanol at 4°C overnight. Next, they were washed with PBS and incubated in the dark with Propidium Iodide (PI)/RNaseA staining solution at 37°C for 30 min. The cell cycle distribution was determined with the fluorescence-activated cell sorter (BD, FACSCantoTMII, USA) using FACSDiva Version 6.1.2 software.

### Cell apoptosis assay

For the apoptosis assay A549 and A549/G^+^ cells were use with trypsin without EDTA to harvest 48 h after drug treatment. Then, the cells were stained with the AV/7-AAD apoptosis detection kit (KeyGEN, China) according to the manufacturer’s instructions. Stained cells were immediately analyzed using a Flow cytometer (BD, FACSCantoTMII, USA). Early (AV^+^/7-AAD^−^) and late (AV^+^/7-AAD^+^) apoptotic cells were included in the determination of cell death. All the data were analyzed by fluorescence-activated cell sorter (BD, FACSCantoTMII, USA) using FACSDiva Version 6.1.2 software.

### Immunofluorescence assay

To obtain the morphologic images of DNA damage foci, cells were seeded at 4 × 10^4^ per well in a 24-well plate containing a glass coverslip in each well and then treated with 0 or 10 μM gemcitabine for 24 h. After treatment, the cells were fixed in 4% tissue cell fixative fluid (Solarbio, China) for 30 min at 4°C and rinsed three times with PBS. The cells were then permeabilized in 0.5% Triton X-100 (Solarbio, China) and incubated with 10% goat serum (Beyotime, China) to block nonspecific antibody binding for 2 h at room temperature. Subsequently, the cells were immunostained with antibodies against phospho-histone H2AX-Ser139 (diluted 1:400, CST, cat. no. 9718, USA) overnight at 4°C. Followed by 1-hour incubation with Dylight 488-Goat Anti-Rabbit IgG (diluted 1:1000, Abbkine, cat. no. A23220, China) in the dark at room temperature. DAPI (4’,6-Diamidino-2’-phenylindole, 10 ng/ml, Solarbio, China) was incubated with cells for 15 min. And the coverslips were washed three times with PBS before mounting. Fluorescence imaging was performed using laser scanning confocal microscopy (Leica TCS SP5; Leica, Germany). The cells containing more than 5 γ-H2AX foci were counted under blinded conditions. That is to say, the observer counts 100 cells in different groups without knowing the grouping, and records cells containing more than 5 γ-H2AX foci in each group.

### Neutral comet assay

Cells were seeded at 6 × 10^4^ per well in a 12-well plate and treated with 0 or 10 μM doses of the drugs the following day. After cells were harvested, PBS was added to re-suspend, blow and mix into single cell suspension. Add 75 μL 1% ordinary melting point agarose as the first layer of gel, then add 75 μL 0.7% low melting point agarose and 10 μL cell suspension as the second layer of gel, last add 75 μL 0.7% low melting point agarose as the third layer of gel. The cells were treated in lysate at 4°C for 3 h, followed by electrophoresis for 20 min, and stained with 0.1 mg/mL PI dye for 10 min under dark conditions. The results were observed under a fluorescence microscope. Under a fluorescence microscope (Olympus, Japan), 100 cells were counted randomly in each sample, and the tail length was measured quantitatively by CASP software (Beijing Baile Liangcheng Technology Co., Ltd, China) to assess DNA strand breaks.

### Statistical analysis

Data were presented as the means ± standard deviation (SD). An unpaired two-tailed t-test or one-way analysis of variance were used with Turkey, Dunnett-T3, or Dunnett post-doc test. *P*  < 0.05 was considered significant. Data analysis was used by SPSS (International Business Machines Corporation, USA) 25.0. Scientific graphing was performed with GraphPad Prism (GraphPad Software Company, USA)8.0.

## Supplementary Material

Supplementary.docx

## Data Availability

The authors hereby confirm that the data supporting the conclusions of this study are included in the manuscript and its supplementary materials.
