# Supplementary material for: CHK1 inhibition overcomes gemcitabine resistance in non-small cell lung cancer cell A549
Source: Mol Cell Oncol. 2025 Apr 9;12(1):2488537. doi: 10.1080/23723556.2025.2488537 (PMC11988257; doi:10.1080/23723556.2025.2488537)
Supplement: Supplementary.docx [file KMCO_A_2488537_SM6680.docx]

Supplementary Content


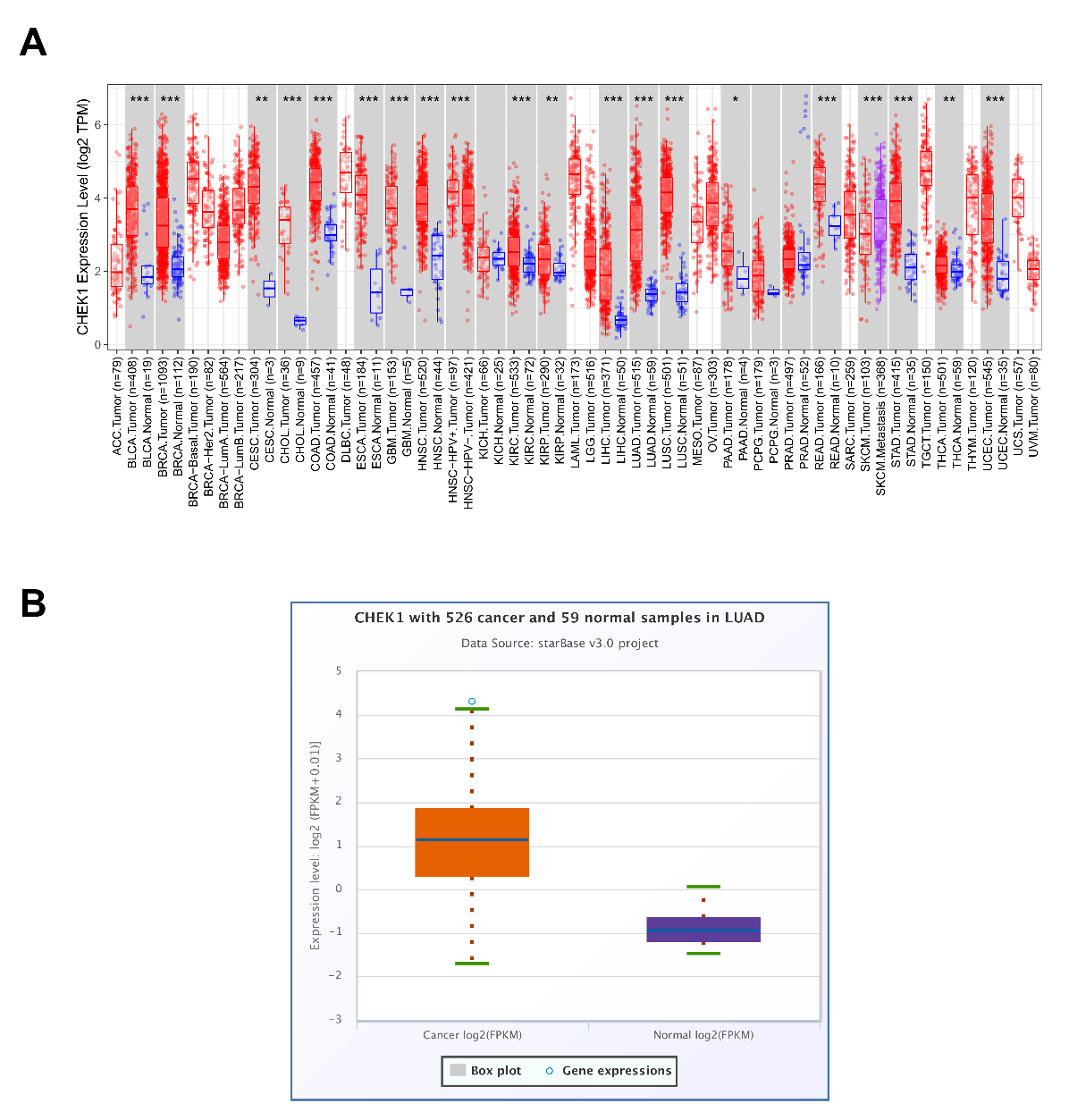


**Figure S1.** Expression of CHK1 in tumors and normal tissues.

**A**: Expression level of CHK1 in tumors and normal tissues; **B**: Expression level of CHK1 in lung adenocarcinoma and normal tissues. ***: *P*<0.001, **: *P*<0.01, *: *P*<0.05.


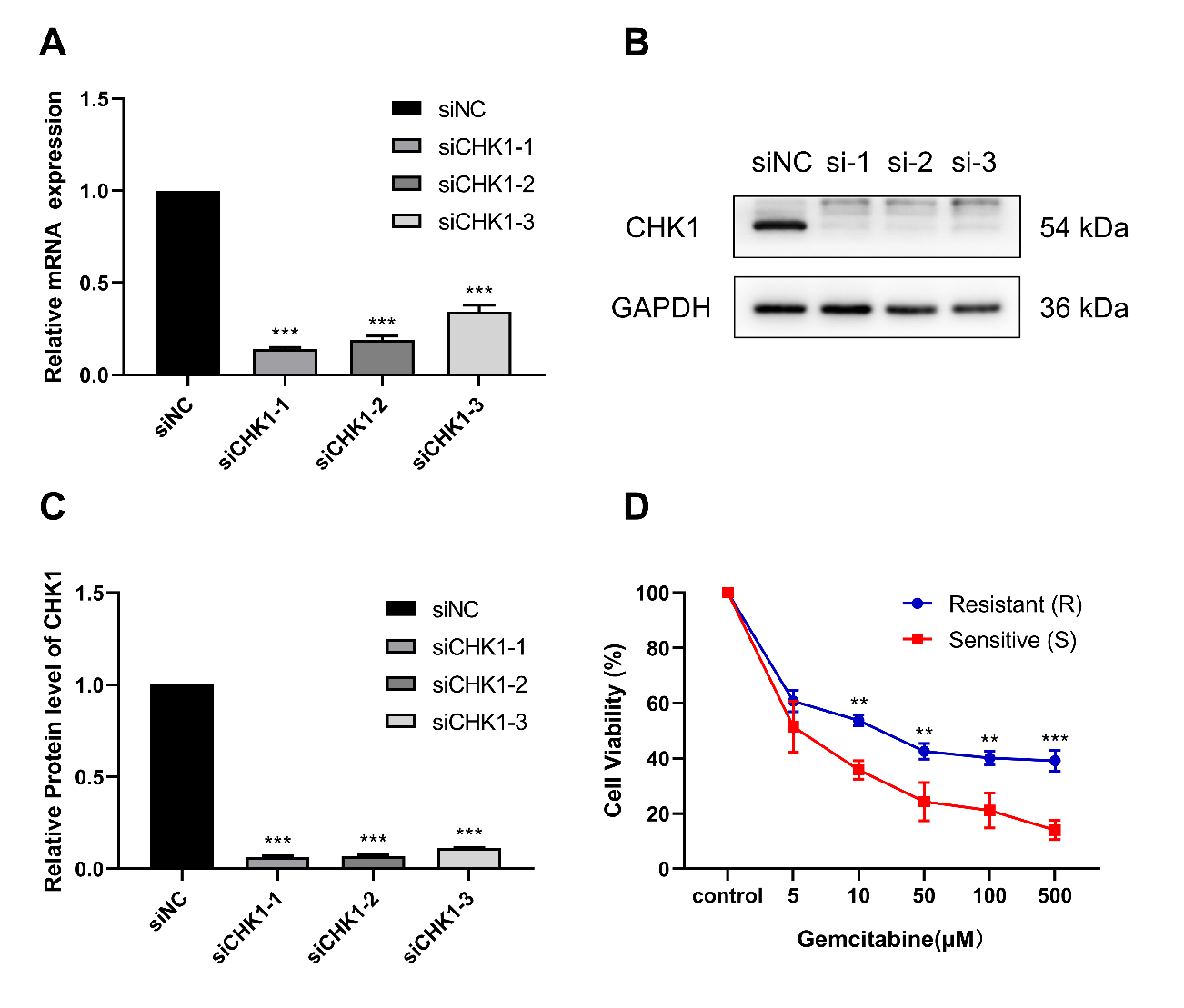


**Figure S2.** **A:** Bar graphs show the difference in CHK1 mRNA levels between the siNC group and the siCHK1 group after siRNA transfection. Compared with the siNC group, ***: *P* < 0.001. **B:** Differences in CHK1 protein levels were evaluated by Western blot between the siNC group and the siCHK1 group after siRNA transfection. GAPDH was used as a loading control. **C:** Bar graphs show the relative protein levels of CHK1 in the siNC group and the siCHK1 group. GAPDH was used as a loading control. Compared with the siNC group, ***: *P* < 0.001. **D:** Cell survival rate of A549 sensitive and resistant strains. Cells were treated with the indicated doses for 72 h, and data are presented as the mean ± SD of three independent experiments. Compared with the siNC group, **: *P* < 0.01; ***: *P* < 0.001.

**Materials and Methods**

Bioinformatics analysis of differential expression of CHK1 in various tumors and normal tissues

We conducted bioinformatics analysis using two databases to obtain differences in expression between tumor tissue and normal tissue. Open the TIMER 2.0 database (http://timer.cistrome.org), choose "Exploration", select the "Gene_De" module, enter the gene name, and analyze the differential expression of CHK1 in tumors and normal tissues. And open the ENCORI database (http://starbase.sysu.edu.cn), select "Pan-Cancer", choose "Gene Differential Expression", enter the gene name, select Lung Adenocarcinoma (LUAD), and analyze the differential expression of CHK1 in lung adenocarcinoma and its normal tissues.
